# Stem cells in genitourinary regeneration

**DOI:** 10.1186/scrt510

**Published:** 2014-10-27

**Authors:** John D Jackson

**Affiliations:** Wake Forest Institute for Regenerative Medicine, Wake Forest School of Medicine, Medical Center Boulevard, Winston-Salem, NC 27157 USA

The demand for treatment options for the genitourinary tract is ever increasing. Disease, congenital malformations, and injury all lead to decreased function of the organs that make up the genitourinary system. Transplantation has been used successfully to replace failed organs. However, as a result of the limited number of transplantable organs and other complications associated with transplantation, regenerative medicine in conjunction with transplantation may be a future alternative [1]. The use of stem cells in the context of regenerative medicine therapies also opens additional treatment options.

In light of these issues, *Stem Cell Research & Therapy* solicited reviews that focused on the use of stem cells for regeneration of four organs within the genitourinary system. Eirin and Lerman [2] examine the use of mesenchymal stem cells in the treatment of chronic kidney disease. Qin, Long, Deng, and Zhang [3] describe the isolation, characterization, and potential use of urine-derived stem cells in the regeneration of bladder. As single cell therapy modalities or combined with biomaterials in tissue engineering strategies, stem cells will play an important role in regeneration of the urinary system in patients with diseased or injured organs.

The other two reviews cover the exciting area of germline stem cells and their use in regeneration of ovaries and testes. Dunlop, Telfer, and Anderson [4] detail the characterization of adult ovary germline stem cells. Sadri-Ardekani and Atala [5] review the identification and expansion of spermatogonial stem cells. Both groups discuss the potential clinical application for these germline stem cells. As these technologies develop, they will be critical in the preservation of fertility in patients undergoing treatments that reduce or eliminate fertility.

We hope that this series of review articles will stimulate interest in stem cell-induced regeneration of the genitourinary system. *Stem Cell Research & Therapy* is an excellent forum for the exchange of ideas and information. We encourage our readers to participate by contributing their thoughts and views in this active research area.

## Note

This article is part of a thematic series on *Stem cells in genitourinary regeneration* edited by John Jackson. Other articles in the series can be found online at http://stemcellres.com/series/genitourinary.
